# An open-source robotic platform that enables automated monitoring of replicate biofilm cultivations using optical coherence tomography

**DOI:** 10.1038/s41522-020-0129-y

**Published:** 2020-04-01

**Authors:** Luisa Gierl, Kasper Stoy, Andrés Faíña, Harald Horn, Michael Wagner

**Affiliations:** 1 Water Chemistry and Water Technology, Engler-Bunte-Institut, Karlsruhe Institute of Technology, Karlsruhe, Germany; 2 Robotics, Evolution and Art Lab, IT University of Copenhagen, Copenhagen, Denmark

**Keywords:** Biological techniques, Biofilms

## Abstract

The paper introduces a fully automated cultivation and monitoring tool to study biofilm development in replicate experiments operated in parallel. To gain a fundamental understanding of the relation between cultivation conditions and biofilm characteristics (e.g., structural, mechanical) a monitoring setup allowing for the standardization of methods is required. Optical coherence tomography (OCT) is an imaging modality ideal for biofilms since it allows for the monitoring of structure in real time. By integrating an OCT device into the open-source robotic platform EvoBot, a fully automated monitoring platform for investigating biofilm development in several flow cells at once was realized. Different positioning scenarios were tested and revealed that the positioning accuracy is within the optical resolution of the OCT. On that account, a reliable and accurate monitoring of biofilm development by means of OCT has become possible. With this robotic platform, reproducible biofilm experiments including a statistical analysis are achievable with only a small investment of operator time. Furthermore, a number of structural parameters calculated within this study confirmed the necessity to perform replicate biofilm cultivations.

## Introduction

Biofilms are microorganisms embedded in a matrix of extracellular polymeric substances^1^. They have several beneficial characteristics which today are of increasing interest, e.g. in cleaning up sewage or in producing platform chemicals, bioplastics or bioelectric currents. In contrast, they e.g., contribute to reducing the efficiency of heat exchangers^2^ or lead to chronic infections of the human body^3^. Hence, biofilm research is trying to understand the development and structure of these aggregates. On that account, methods are required, which capture the dynamics of biofilm growth or detachment. Lab-experiments often apply flow cell setups to improve our understanding of structure development and dynamics^4–6^; for example the effects of nutrient, substrate, and hydrodynamic conditions^7–9^. Due to restrictions regarding human resources and time, experiments are mostly conducted with a low number of replicates (e.g., *n* ≤ 2)^10–12^. Hence, the determined population does not allow for a statistical analysis. To tackle these challenges, combining a fast imaging modality to assess the biofilm structure with a positioning device allowing for the investigation/monitoring of several flow cells running in parallel would be preferable.

Since a commercial fully automated monitoring setup was not available, a system combining reproducible in situ visualization of biofilms by means of optical coherence tomography (OCT) and automated accurate positioning of the OCT probe was developed.

Recently, OCT has been widely applied in biofilm research e.g. for analyzing biofilm structure and deformation in flow cells as well as in membrane filtration or in the evaluation of cleaning procedures^9,13–15^. The increasing number of scientific publications employing OCT^16,17^ shows its advantages of being non-invasive and providing real-time information^15–17^. Moreover, this imaging technique fulfills the necessity for high-resolution biofilm structure identification (for more information see Materials section).

In the present study, the weight of the OCT scanning probe (approx. 1.5 kg) appears to be the bottleneck in designing a fully automated and accurate positioning and monitoring system. However, to avoid sloughing of the biofilms, moving the OCT probe should be better and less time consuming than moving the flow cells. Currently, a couple of devices are commercially available, which might be combined with OCT imaging. 3D printers are of interest as they are addressable by scripting, cheap and have a supporting user community. Fitzsimmons et al.^18^ recently generated a low-cost bio printer that is conceived to be modular and open source^18^. Users have the convenience to assemble the printers and accomplish iterative enhancements on their own. In contrast, the construction is not designed for moving heavy parts as an OCT scanning probe.

From a practical point of view CNC systems like the Stepcraft DIY CNC (Stepcraft GmbH & Co. KG, Menden, Germany) should be well-suited to be combined with an OCT as those machines handle heavy loads. They are often cheaper than 3D (bio) printers, robust and accurate. However, 3D printers and CNC systems are delivered with specific software and it still takes much efforts converting hard- and software from 3D printing or routing/milling applications into an automated OCT-based monitoring setup for biofilms. However, if one is willing to accept these challenges, Depetris et al.^19^ recently presented their approach on a modified CNC machine^19^.

**Fig. 1:**
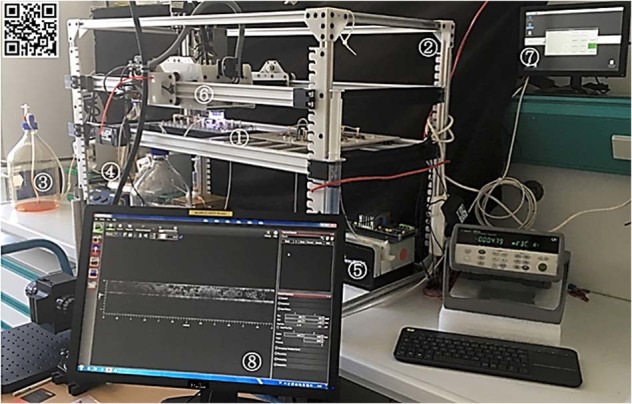
Photograph of the monitoring and screening setup. Biofilm cultivations are performed in flow cells installed on the experimental layer (1) of the EvoBot (2). Additional equipment such as cultivation media (3), magnetic stirrers (4) and peristaltic pumps (5) are located below the experimental layer. The cultivation media is pumped through the flow cells without recirculation. The OCT scanning probe is installed in the OCT module (6) uniquely provided by the EvoBot platform. A Raspberry Pi (7) controls the EvoBot platform through a graphical user interface and a command line tool. Positioning of the OCT module is either performed manually or automated using scripting. By means of the ThorImage OCT software (8), structural changes in biofilms can be logged directly while running an experiment. The QR code links a video clip showing the EvoBot in action.

**Fig. 2:**
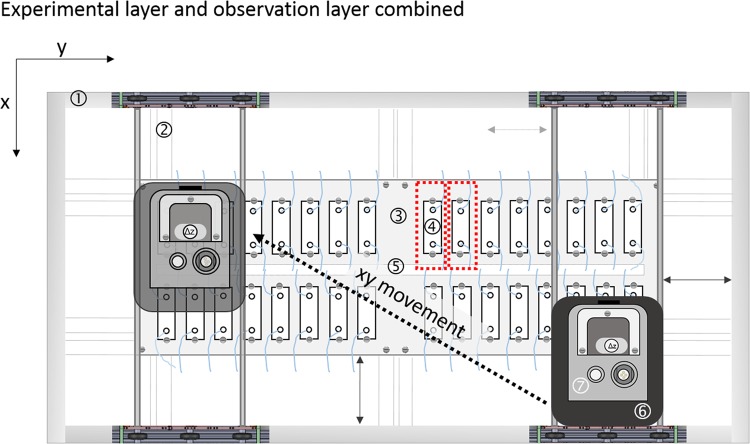
Experimental layer and setup of the flow cells. The outer frame/foot print (*A*_F_ = 60 × 90 cm^2^ (1)) and mounting supports (2) fixate and stabilize the experimental layer. Within the imaging area (*A*_I_ = 27 × 57 cm^2^ (3)) up to 32 flow cells (*A*_FC_ = 2.6 × 7.6 cm^2^ (4)) can be fixed in place with screws. Sufficient space (5) for tubing (blue lines) is provided. The actuator head (6) is used to position the OCT scanning probe (7). The red dashed lines illustrate the general area needed to mount the flow cells including tubing, screws and space in between adjacent flow cells (*A*_GFC_ = 14 × 3.5 cm^2^).

A robotic platform called EvoBot, which was created within the EVOBLISS project, has been available for a few years (https://blogit.itu.dk/evoblissproject/)^20^. The EvoBot platform is a modular open-source setup (soft- and hardware) suitable to perform fully automated biofilm cultivations including the option for biofilm visualization by means of OCT. The advantages of the EvoBot platform compared to the other robotic equipment are manifold. The mechanical design (e.g., size of working space) is flexible and an application programming interface (API) provides a user-friendly base for developing own, optimized software. Most importantly, the EvoBot platform already includes an OCT module (hardware) as well as ready-to-use software to control its positioning (see Fig. 1). Additional modules such as USB cameras, syringes and extruders can also be installed extending the experimental degree of freedom. The experimental layer has a generous usable area of 90 × 60 cm^2^ so that several flow cells containing biofilms are easy to install, operate and process (see Fig. 2). The experimental layer also provides sufficient space for arranging necessary experimental devices such as magnetic stirrers, pumps and vessels for the cultivation medium. On these grounds, the EvoBot robotic platform was selected for monitoring biofilm development in several flow cells operated simultaneously by means of OCT. The approach was further used to approve the necessity of replicate biofilm cultivations in order to retrieve valid and reliable parameters describing biofilm structure.

## Results

### Positioning accuracy

Reliability and representativeness of structural parameters depend on the accuracy of the positioning of the OCT scanning probe. It is required that the positioning accuracy is equal or smaller than the optical resolution of the OCT. Hence, a valid and reliable treatment of the data should be possible if Δ*x*_CoM_ and Δ*y*_CoM_ ≤ 8 µm. Therefore, 100 independent images of the target (red printed circle with a diameter = 1 mm) were captured and analyzed as described in subsection Methods. Statistical distribution is described below whereas Table 1 in Supporting Information (SI) summarizes the derived information. Grubbs’ tests revealed no outliers. Hence, values are calculated based on the community of *N* = *n* = 100 (per positioning condition).

The first positioning condition P1 with simultaneous movement along the *x*- and *y*-axis represents the default setting of the EvoBot platform. Starting from the origin (fixed position at 0,0,0), it was possible to image the target with high accuracy. On average the deviation Δ*x*_CoM_ and Δ*x*_CoM_ from the mean coordinates $\bar x_{{\rm{CoM}}}$ and $\bar y_{{\rm{CoM}}}$ is almost zero, with all of the data for the *x*-axis varying between −4.3 µm–3.6 µm from the mean $\bar x_{{\rm{CoM}}}$. Positioning on the *y*-axis was less accurate (−8 µm ≤ Δ*y*_CoM_ ≤ 10.7 µm). Positioning scenario P4 revealed similar results. However, P4 showed several values for Δ*x*_CoM_ and Δ*y*_CoM_ outside the 1.5 IQR (Interquartile Range), which seemed to be random.

Generally, for P2 (starting from random positions; see SI Fig. 1) variations from the mean CoM were higher compared to P1, pointing a deviation |Δ*x*_CoM_|of up to 36.9 µm and |Δ*y*_CoM_| up to 15.3 µm including several values outside 1.5 IQR. Higher deviations also appeared for P3, where movement from the fixed origin started on the *x*-axis, followed by the *y*-axis.

In summary, experiments P3 and P4 revealed that non-diagonal positioning leads to less accurate positioning of the actuator head. Nevertheless, with each positioning scheme it was possible to realize a positioning accuracy of |Δ*x*_CoM_| and |Δ*y*_CoM_| ≤ 8.6 µm for at least 75% of the estimations (*N* = 100).

Due to the location of the 24 flow cells under investigation, it was required to combine all four positioning schemes.

### Monitoring biofilm development

To assess the usability of the EvoBot platform in combination with OCT imaging for replicated investigations, simplified proof-of-concept biofilm experiments were conducted. The EvoBot platform was operated without user interaction. In total 24 flow cells were operated in parallel without interfering with each other. The necessity for independent replicate cultivations was evaluated by calculation and statistical treatment of several structural parameters as described in the Methods section.

**Fig. 3:**
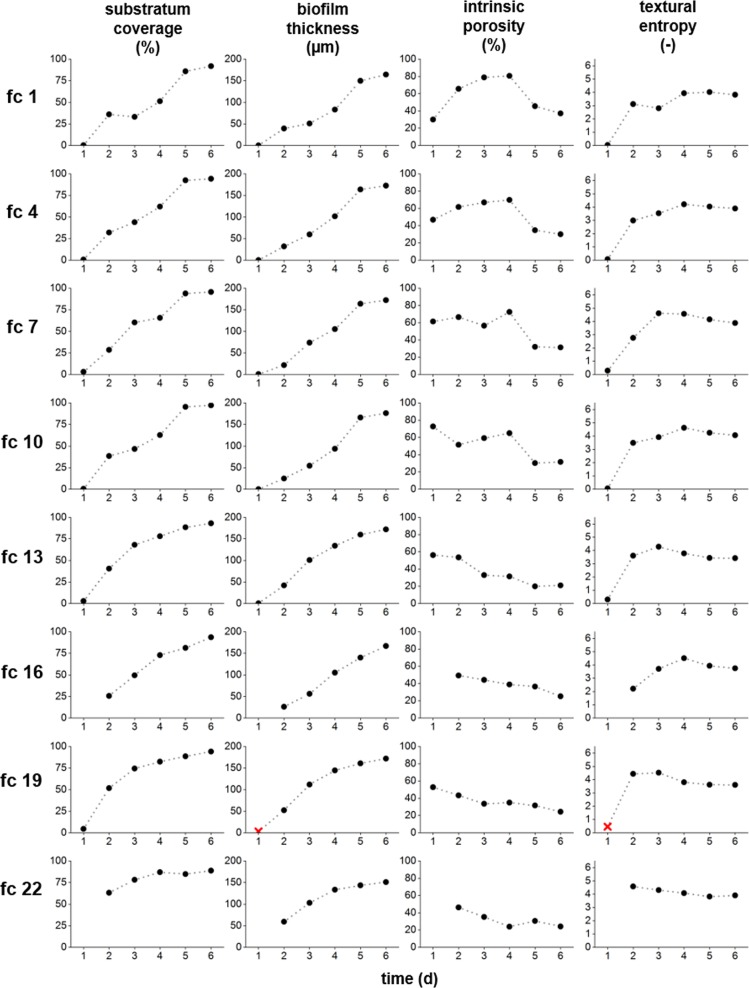
Development of structural biofilm parameters. Substratum coverage SC, mean biofilm thickness $\bar L_F$, intrinsic biofilm porosity Φ_intrinsic_ and textural entropy TE illustrated for eight out of 24 biofilms over the course of the experiment (six days). Red crosses (markers) indicate outliers in flow cell 19. Missing values are due to visualization artifacts and were excluded from further calculations.

Figure 3 depicts the development of the structural parameters substratum coverage (SC) mean biofilm thickness $\left( {\bar L_F} \right)$, intrinsic biofilm porosity (Φintrinsic) and textural entropy (TE) of eight biofilms over the course of the experiment (six days). Therefore, every third flow cell out of *N* = 24 samples is displayed. Flow cell no. 1 (fc 1) has randomly been selected as initial flow cell.

**Fig. 4:**
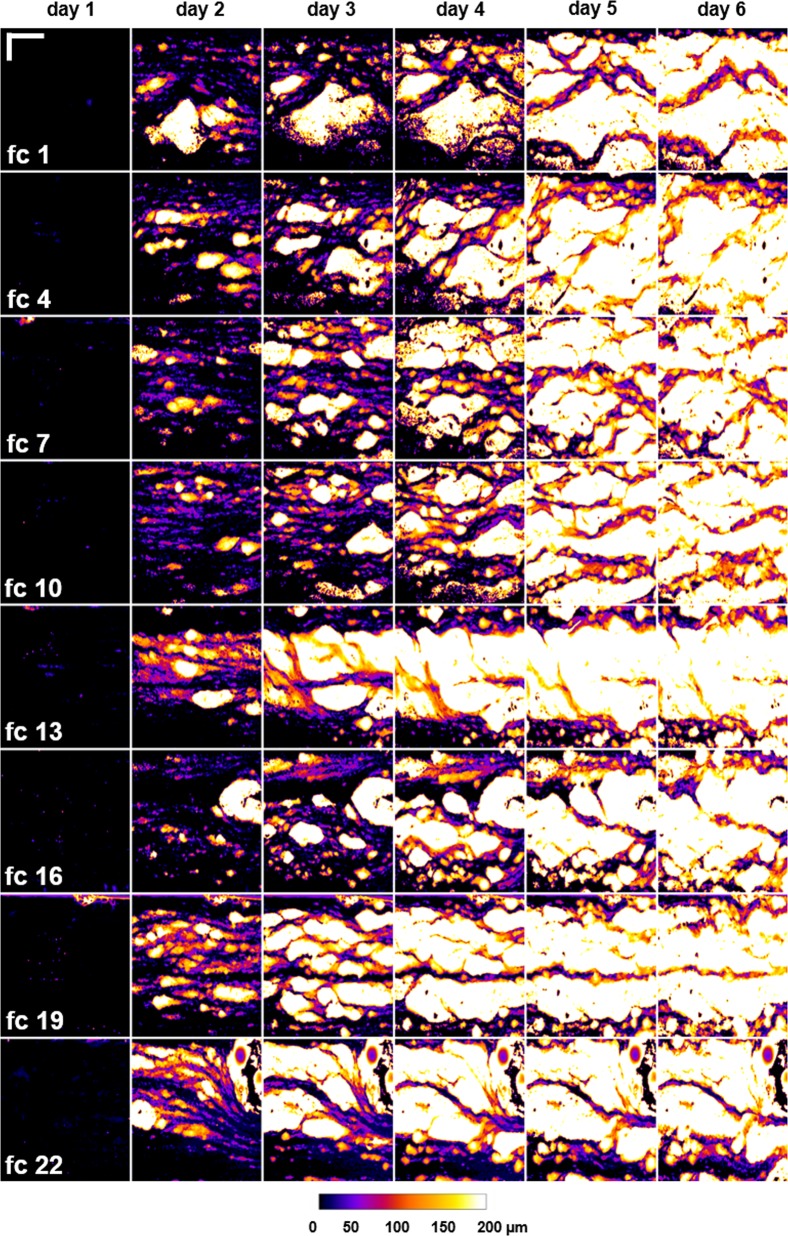
Biofilm topographies (height map, bulk-biofilm interface) over the course of the experiment of the stated flow cells displaying the biofilm development. Image size is 5 × 7 mm^2^. The scale bar (*x* = 2 mm; *y* = 1 mm) as well as the calibration bar (biofilm height in the *z*-direction in µm) are given. Flow from left to right.

SC thereby illustrates the coverage of the substratum with biofilm. $\bar L_F$ describes the growth and accumulation of biofilm in terms of biofilm thickness calculated from the bottom to the top of the flow channel (z-direction). Φ_intrinsic_ characterizes the amount of voids and cavities within the biofilm and correlates it to the amount of biomass detected. TE quantifies the heterogeneity of the bulk-biofilm interface. In Fig. 4 the topography of the bulk-biofilm interface is shown (height maps) depicting the structure of the biofilm.

Figure 3 therefore reveals an increase of biomass accumulating at the substratum (SC) in all flow cells over the course of the experiment, starting from an empty channel with zero coverage until almost 100% of the substratum are covered with biofilm. These results are supported by a constant increase of $\bar L_F$, where the biofilm thickness reaches 200 µm at the end of the experiment (compare Figs. 3 and 4). Hence, a similar trend for both parameters can be estimated. As seen from Fig. 4 biofilms develop locally forming patches which within 6 days cover the entire imaging area. However, between days 0 and 3 a non-uniform biofilm distribution has been observed. In comparison, Φ_intrinsic_ shows diverging results for the biofilms illustrated. Here, biofilm porosity can vary up to 50% as seen at day 1 of the cultivation. Flow cells 1, 4, 7, and 10 depict an increase of biofilm porosity until day 4 with a decrease of porosity at days 5 and 6. In contrast, flow cells 13, 16, 19, and 22 reveal a consistent decrease of Φ_intrinsic_ from 40 to 60% at day 1 to Φ_intrinsic_ = 20% at day 6. Reduction in biofilm porosity (Φ_intrinsic_) can be explained by closure of void spaces. Thus, Φ_intrinsic_ reached its maximum at day 4 and decreased afterwards.

Furthermore, an increase in *TE* defines an increase in heterogeneity. Such an increase was observed for all flow cells from day 1 (TE = 0) to day 2 (TE = 2–5). These results are disclosed by Fig. 4. Biofilm colonies are growing divergently, forming smaller and thicker as well as shorter and longer patches; similarly depicted in the development of Φ_intrinsic_. Values of the parameters Φ_intrinsic_ and TE differ whereas values for SC and $\bar L_F$ seem to be similar for all flow cells. More detailed information is provided in SI Figs. 2–7 showing the course of each parameter for all 24 flow cells analyzed.

**Fig. 5:**
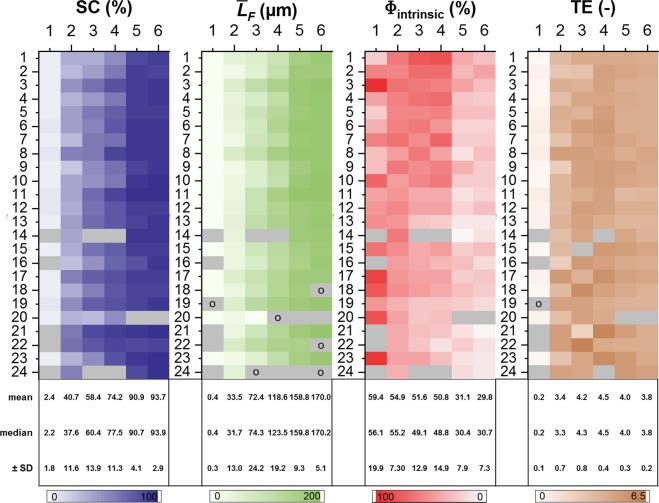
Heat maps showing the development of the structural parameters. SC = substratum coverage; $\bar L_F$ = mean biofilm thickness; Φ_intrinsic_ = intrinsic biofilm porosity; TE = textural entropy. Means, medians, and standard deviations (SD) are given. Gray cells define missing values (e.g. caused by imaging artifacts); “o” denotes outliers.

Figures 3 and 4 only show a subset of flow cells and might thus give an erroneous picture of the stated parameters. To show the necessity for replicate experiments, Fig. 5 provides a more comprehensive view due to the larger number of flow cells (*N* = 24).

As known from statistical data analysis, the accuracy of parameters increases with the number of replicates^21,22^. This is reflected for SC and $\bar L_F$ on day 1 in Fig. 5. On the first day, mean and median values are similar: $\bar x$_SC_ = 2.4% and $\tilde x$_SC_ = 2.2% and $\bar x_{\bar L_F}$ = 0.4 µm and $\tilde x_{\bar L_F}$ = 0.4 µm, respectively. Additionally, uniform color intensities in the heat maps indicate a primarily homogenous distribution of parameter values for *SC* as well as $\bar L_F$ (*n* = 24) at the beginning of the experiment. With the duration of the experiment, mainly at day 3, distribution of color intensities start to spread and SD reaches maximum values (±SD_*SC*_ = 13.9%; ±${\mathrm{SD}}_{\bar L_F}$ = 24.2 µm). At day 5 of the experiment, means approximate medians and SD reaches values one third of that calculated for day 3. This observation is in agreement with biofilm development as shown in Fig. 4 (SC > 90% and $\bar L_F$ up to 160 µm at day 5).

A different picture is drawn for the structural parameter Φ_intrinsic_. Here, day 1 reveals the widest spectrum of color shading reflected by the highest ${\mathrm{SD}}_{\Phi _{{\rm{intrinsic}}}}$ of ± 19.9% during the entire cultivation. Furthermore, ${\mathrm{SD}}_{\Phi _{{\rm{intrinsic}}}}$ = 7.9% is still high at day 5. This value almost equals one fourth of the mean $\bar x_{\Phi _{{\rm{intrinsic}}}}$ = 31.1% and median $\tilde x_{\Phi _{{\rm{intrinsic}}}}$.

Lastly, heat maps of TE in Fig. 5 illustrate a more homogenous color intensity distribution compared to *SC* and Φ_intrinsic_. This is opposing to Fig. 3 which revealed diverging diagrams for TE at days 2 and 4. Any contradiction between presented results might be caused due to the fact that Fig. 3 only shows a subset of eight flow cells.

## Discussion

Due to the optical characteristics of the used GANYMEDE I OCT imaging system equipped with an LSM03/LK3 objective lens, the required positioning accuracy in the *x*- and *y*-direction needs to be ≤8 µm (optical resolution). If so, inaccurate positioning is not resolved. Results of the statistical evaluation of the EvoBot platform demonstrated the capability of the entire monitoring setup (EvoBot + OCT) to investigate biofilm structure development and quantitative assessment. With the current setup, 75% (e.g., IQR) of all movements performed have a maximum inaccuracy of |8.6 µm|.

The determined positioning accuracy was even better than proposed by the developers of the EvoBot platform^20^. The EvoBot platform thus offers accurate positioning of the OCT scanning probe and allows for applying OCT as an automated monitoring tool. The most accurate positioning of the OCT probe is achieved if the OCT probe is homed before moving to a specific location. This will be at the expense of time and might thus not always be the scenario of choice.

However, positioning was more inaccurate on the *y*-axis than on the *x*-axis. Deviations between the *x*- and *y*-axis might be related to the geometry of the platform. The EvoBot platform used in this study has a working area of 90×60 cm^2^. Thus, there is more travel distance on the *y*-axis possibly leading to larger deviations as more deviations per step are summing up. To improve the resolution further, micro-stepping modes could be changed from 1/16 to 1/32 to reduce mechanical noise and resonance problems^23^. Furthermore, stepper motors with encoders could be used to avoid losing of steps. This might become necessary if higher micro stepping (e.g., 1/32) is applied. For larger masses installed into the payload module, stepper motors with a higher torque might be required for accurate positioning. Taken these issues into account, the EvoBot platform was operated in 1/16 micro-stepping mode as compromise. There are other tweaking options (e.g., higher resolution stepper motors, acceleration and deceleration settings, timing belt resolution, etc.) available. However, in this study the chosen options fulfill our needs for applying the EvoBot robotic platform for the structural characterization of biofilms by means of OCT imaging. Moreover, moving the OCT scanning probe between adjacent imaging positions merely took 3–5 s which emphasizes the applicability of the EvoBot platform since the acquisition of OCT datasets is more time consuming.

Biofilm development was characterized and quantified calculating the following structural parameters: SC, mean biofilm thickness ($\bar L_F$), intrinsic biofilm porosity (Φ_intrinsic_) and TE. Differences in such parameters are valuable for correlating fluid-structure interactions, mass-transport dynamics as well as variations between biofilm species and/or cultivation procedures. Thereby, the parameters can even predict which culture conditions to apply for maintaining (beneficial) or get rid of (useless/harmful) biofilms.

In the present study, major differences within stated parameters develop in the mid of the experiment (day 2–4). Nevertheless, biofilms develop with a steady increase of the mean biofilm thickness $\bar L_F$ (and a steady decrease of intrinsic porosity Φintrinsic (see Fig. 5).

As mentioned before and supported by various studies^10–12^, small numbers of replicates (*n* ≤ 2) may lead to erroneous conclusions and misinterpretation of results. Importantly, the possible incorrectness of results is not observable, because the “correct” value of a parameter is statistically not approached. Figure 5 shows the variation of the selected structural parameters over time and among the community of 24 independent biofilm cultivations. In general, with ongoing cultivation the values of the structural parameters equalize between the different biofilm cultivations (flow cells). However, the younger the biofilm the greater the differences between flow cells. Additionally, outliers occur unpredictably. Again, within a reduced community outliers might not even been identified as such. As an in-detail example, it can be seen in Fig. 5 for Φ_intrinsic_ that flow cells 1, 2, 3 and 4 deliver completely different color-coded values at day 1. In these cases the ‘real’ biofilm porosity Φ_intrinsic_ [Φ_intrinsic_ (*n* = 1,2,3…) ≠ Φ_intrinsic_ (*n* = ∞) ≠ *μ* (Φ_intrinsic_))] obtained from *n* = 2, *n* = 3 and *n* = 4 might be over- or underestimated due to missing replicates. This fact has been addressed by Majewsky et al.^24^ They mentioned that although it is well known by researchers that with increasing number of replicates the accuracy of the result becomes more correct, only a few conduct replicate experiments. Often one reason are experimental limitations.

Although Ahimou et al.^25^ performed repeated experiments with *n* = 6, their results still show deviations of mean biofilm thickness of approximately ± 200 µm (≙ ±17%) for 12 days old biofilms^25^. This may mean that (i) the experimental conditions cause biofilm structures with such high variations of the mean biofilm thickness or (ii) six replicates are still insufficient to determine the mean biofilm thickness correctly.

Turonova et al. (2012) for example used three replicates in their study having up to ±100 µm standard deviation (≙ ±29%) in maximum biofilm height^26^. Similar results have been reported by Bester et al.^27^ using duplicates, Koseki et al.^28^ using up to *n* = 12 replicates and Sauer et al.^29^ using at least triplicates^27–29^.

Depending on the parameter to estimate, even duplicates or triplicates may lead to small standard deviations due to culture conditions or simply by chance. For example, SC at day 2 for flow cells 8, 17, and 19 does not vary a lot (see Fig. 5). Similar observations were made for $\bar L_F$ (flow cells 2 and 4 at day 4) or intrinsic porosity Φ_intrinsic_ (day 4, flow cells 16 and 23), respectively. However, the dataset obtained with 24 flow cells seems to indicate that the true value of these parameters lies elsewhere. Such erroneous estimations of structural parameters of the biofilm could have important consequences. Indeed, since physical structure determines the interaction between biofilm and its environment^30^, a miscalculation due to small sample sizes could lead to misinterpretation of the results. Examples of mistaken conclusions that might be drawn from such miscalculations include the percentage of effectiveness of an anti-biofilm agent, the choice of optimization parameters of a trickling filter in a waste water treatment plant^31^, or the best solution to avoid biofilm-induced blocking and damage in industrial settings.

By employing the developed monitoring setup (EvoBot + OCT) experimental limitations can be narrowed down as a high number of micro- and or mini-fluidic flow cells (up to 32 slide-sized flow cells) can be mounted, operated, and monitored on-line and fully automated.

The presented study showed the importance of quality control to reduce contradicting reports on biofilm parameters. Simultaneously performed replicates of *N* = 24 demonstrated that differences between biofilms grown in flow cells under the same condition may or may not occur for a specific parameter. According to what is measured, it is necessary to adapt the number of replicates to the statement of a research study. Generally, larger sample sizes increase the statistical power of a survey. This was already mentioned and illustrated in detail by several examples in the publication of Wilson VanVoorhis and Morgan^32^.

In this study a monitoring setup for biofilms was developed. It employs the EvoBot robotic platform for positioning an OCT scanning probe in order to visualize the biofilm structure within micro- and mini-fluidic flow cells. The setup is automated and facilitates replicate experiments. Results clearly confirm the need for replicate cultivations/experiments in biofilm research. Replicates allow for a statistical treatment of experimental results drawing valid conclusions about for example the effect of the mean flow velocity on the biofilm structure. Main achievements of this study are:

- highly accurate and reliable positioning of the OCT scanning probe,
- fully automated and non-invasive monitoring of biofilm development,
- high-throughput screening and thus saving of time,
- statistically accurate conclusions about monitored biofilm development (e.g., for flow cell experiments).

With the advantages of being low-cost, open-source and user-friendly, the EvoBot robotic platform is available to everyone.

## Methods

### EvoBot robotic platform

The monitoring setup consists of the EvoBot robotic platform (for further information see: https://blogit.itu.dk/evoblissproject/ or https://bitbucket.org/afaina/evobliss-software/wiki/Home). Figure 1 shows a photograph of the entire lab-scale system used in this study.

Briefly, the EvoBot platform is composed of a structural aluminum frame and two working layers. The top layer is the actuator layer. Implemented on this layer is the actuator head, which can move in the horizontal (*x*, *y*) plane and carry several modules (e.g., syringe modules, OCT module). A heavy payload module (OCT module) is available to position the OCT scanning probe in all spatial directions. Stepper motors assure a smooth and precise positioning. On the EvoBot platform used in this study, up to 32 slide-sized flow cells (76 × 26 mm^2^) can be assembled and installed on the experimental layer located beneath the actuator layer (see Fig. 2).

Movement of the actuator head is controlled by an Arduino Mega microcontroller board (Arduino S.r.l., Ivrea, Italy) attached to a RAMPS shield (version 1.4, Pololu electronics, Massachusetts, USA). The Arduino Mega operates a custom Marlin firmware developed within the EVOBLISS project. It interfaces with Python scripting (Python 2.7, Python Software Foundation, USA) through an user-friendly API providing simple commands for positioning the OCT module/OCT scanning probe in *x*, *y*, and *z*. Scripting and controlling is performed on a low-cost single-board computer (Raspberry Pi 3B, rev. 1.2, Raspberry Pi Foundation, GB) running Raspbian.

Acquisition of OCT C-scans (3D datasets) is performed by a custom-made command line interface (CLI) for the Thorlabs software development kit (SDK). The Raspberry Pi is connected to the OCT controlling computer via a local area network connection to trigger biofilm visualization by calling the execution of the OCT CLI (separate Python script). Altogether, this enables the automated visualization of biofilm by means of OCT without sloughing events due to unmounting and movement of the flow cells (see Figs. 1 and 2).

Besides command line based operation/control, a graphical user interface (GUI) offers manual handling of the robot and acquisition of OCT scans, leading to the same image result as automatically acquired.

### Statistical evaluation of the robotics’ positioning accuracy

Positioning tests were performed to determine the positioning accuracy of the robotic platform. For these tests an USB microscope with an image size of 1600 px × 1200 px (field of view = 6.7 × 5.0 mm^2^) was mounted to the heavy payload module of the EvoBot platform. The USB microscope captured a printed circle (red colored; diameter = 1 mm; referred to as target) at a fixed location (x/y/z) on the experimental layer. Four different positioning strategies P1 to P4 were tested to assess the effect of movement control (e.g., directionality) of the actuator head on the position accuracy. Strategies are illustrated in Supplementary Information Fig. 1a, b.

Acquired images were automatically analyzed using the open-source software Fiji^33,34^. By using an in-house macro, images were analyzed for the location of the target (red printed circle). Image post-processing is depicted in SI Fig. 1c. Firstly, a mean filter with a radius of 2 px was applied and images were converted to 8-bit grayscale. Through binarization the target (foreground) was separated from the background using the converting to mask plugin (threshold = 120). With the use of the built-in plugin (“analyze particles”), artefacts (e.g., dust particles) were excluded and the Center of Mass (CoM) of the target was calculated. Simple geometric shapes as the analyzed circle have their center of mass at the centroid that is also described as the midpoint of an object^35^. The mean values $\bar x_{{\rm{CoM}}}$ and $\bar y_{{\rm{CoM}}}$ of all midpoints were calculated and subtracted from individual measurements *x*_CoM,*i*_ and *y*_CoM,*i*_ as follows:

1 $$\Delta x_{{\rm{CoM}}} = x_{{\rm{CoM}},i} - \bar x_{{\rm{CoM}}}$$

2 $$\Delta y_{{\rm{CoM}}} = y_{{\rm{CoM}},i} - \bar y_{{\rm{CoM}}}$$

where Δ*x*_CoM_ and Δ*y*_CoM_ denote the deviation from the mean CoM in the *x*- and *y*-dimension. Measurements are in µm.

The higher the values of Δ*x*_CoM_ and Δ*y*_CoM_, respectively, the less the positioning accuracy.

### Biofilm cultivation

Biofilms were cultivated in custom-made flow cells composed of sticky-slides (sticky-Slide I 0.4 Luer, ibidi GmbH, Martinsried, Germany) glued to PVC slides (substrata) with a thickness of 2 mm. The sticky-slides are made from transparent plastic and serve as the cover of the flow cell forming a flow channel in the size of 50 × 5 × 0.45 mm^3^ (length × width × height, thickness of the sticky-slide = 1 mm). 24 flow cells were operated in parallel at a mean flow velocity of *u* = 6 mm/s (volumetric flow rate *Q* = 0.81 mL/min).

Flow cells were inoculated with *Bacillus subtilis* pre-cultures grown at 37 °C overnight in Luria Broth (LB) medium. Cells were grown to exponential phase; 10 mL of these cultures together with a minimal salts glycerol medium (MSGM) in a mixture ratio of 1:500 were used as inoculation solution. The cultivation medium was adapted from Wang et al.^36^ and contained (concentration in mg/L):^36^ CaCl_2_ · 2 H_2_O (110), MgCl_2_ · 6 H_2_O (410), glycerol (5), L-tryptophane (5), L-phenylalanine (5), MnCl_2_ · 4 H_2_O (9.9), FeCl_2_ · 4 H_2_O (2.5), ZnCl_2_ (0.136) and thiamin hydrochloride (0.674). The solution was phosphate buffered to pH = 6.8 with 0.5 M sodium–potassium–phosphate buffer.

Flow cells were flushed with the inoculum for 15 min. Afterwards flow was stopped for 1 h giving bacteria the possibility to settle. Then biofilm cultivation started in flow-through mode at a mean flow velocity of 6 mm/s. Biofilm development 25 mm downstream the inlet was monitored daily for six consecutive days by means of OCT using the EvoBot platform.

### Monitoring biofilm development by means of OCT

OCT is a 3D visualization technique with a high penetration depth in translucent tissues such as biofilms with µm-resolution^9^. OCT allows for the non-invasive, real-time imaging of the mesoscopic biofilm structure as demonstrated by various authors^15,37^.

Briefly, OCT measures a point reflection signal from a given sample and generates a depth-resolved intensity profile along the optical axis (*z*-direction, here: height of the flow channel). Several of these so-called A-scans summarize to a B-scan in lateral direction. Therefore, a B-Scan is a cross-sectional image in the *xz*-plane, creating a side view along the flow channel length. To perform volumetric (3D) scans of biofilms, it is necessary to create a C-scan by acquiring adjacent B-scans^15^.

A spectral domain tomograph (GANYMEDE I, Thorlabs GmbH, Dachau, Germany) with an optical resolution of 8 × 8 × 2.1 µm^3^ (*x* × *y* × *z*, LSM03 objective lens) in water (*n* = 1.33) was used to monitor biofilm development. By generating 2D and 3D datasets within seconds to minutes, biofilms in all 24 flow cells installed on the experimental layer were visualized within 30 min. OCT autocorrelation images (A-scan averaging = 3) with a size of 7 × 6 × 1 mm^3^ were automatically acquired daily starting at 8 a.m. using the command line interface.

Image post-processing included the calculation of structural biofilm parameters. OCT datasets were cropped to a volume of 7 × 5 × 0.45 mm^3^. A mean filter with a radius of 2 px was applied and binary datasets were generated using Fiji. SC, mean biofilm thickness $\bar L_F$, intrinsic biofilm porosity Φ_intrinsic_, TE and global biofilm porosity Φ_global_ were calculated from binary datasets according to Blauert et al.^9^, Wagner and Horn^15^ and by use of the MiToBo plugin (Fiji) for biofilms^9,15,38^. In-house macros were used to render topographic representations of OCT C-scans (e.g., height maps representing the bulk-biofilm interface)^15^.

An overview of additional structural parameters is given in Yang et al.^39^, Beyenal et al.^40^ as well as in Wagner and Horn^15,39,40^.

The chosen structural parameters were calculated as follows.

Mean biofilm thickness $\bar L_F$ was calculated from the substratum of the flow cell to a point in the bulk-biofilm interphase (z-direction) using Eq. (3):

3 $$\bar L_F = \frac{1}{N}\mathop {\sum}\limits_{i = 1}^N {L_{F,i}}$$

where *L*_*F*,*i*_ is a local biofilm thickness measurement at location *i* and *N* equals the number of thickness measurements (if a complete C-scan is analyzed, *N* is equal to the number of A-scans)^15^.

Proper calculation is dependent on correct selection of the voxel/pixel height Δ*z*, which depends on the refractive index *n* of the medium as shown in Eq. (4):

4 $$\Delta z = \frac{{\Delta z_{air}}}{n}$$

thereby Δ*z* being the effective axial resolution in this medium and Δ*z*_air_ being the axial resolution of the device in air (*n* = 1).

Once the locations *i* are determined to their positions in the z-stack (Δ*z*) and set in the binary image (0, 255) it is possible to calculate *L*_*F*,*i*_ (Eq. 5):

5 $$L_{F,i} = \frac{{i \cdot \Delta z}}{{255}}$$

SC is calculated from the Maximum Intensity Projection images (MIPs) in the *xy*-direction.

Therefore, all pixels with a pixel value ≥ 1 in the histogram are defined as biomass growing on the substratum and all pixel values = 0 are defined as bottom/background.

All color-coded pixels are then counted and subtracted from the whole number of pixels available in the image as visual from Eq. (6):

6 $${\rm{SC}} = \frac{{A_{{\rm{biofilm}}}}}{{A_{{\rm{biofilm}}} + A_{{\rm{background}}}}} \cdot 100$$

where *A*_biofilm_ determines the area covered with biofilm and *A*_background_ is the bare area (in comparison: surface coverage is calculated from each slice in a binary z-stack, where a mean is generated similar to $\bar L_F$).

Intrinsic porosity Φ_intrinsic_ is calculated in the *xz*-direction of the flow cell (longitudinal section) and according to Blauert et al.^9^.

In the case of Blauert et al.^9^ and the study performed here background signals outside the structure were excluded. By use of an in in-house macro, the structure (i), voids within the structure (ii) and the background (iii) were automatically set to three different thresholds (150, 50, 0) and computed afterwards as explained in Eq. (7):

7 $$\Phi _{{\rm{intrinsic}}} = \frac{{A_{{\rm{voids}}}}}{{A_{{\rm{biofilm}}} + A_{{\rm{voids}}}}} \cdot 100\;[\% ]$$

*A* thereby indicates the area covered with either biofilm or voids.

For calculating TE, MIPs were analyzed by Eq. (8) using the Fiji MiToBo plugin generated by Möller et al.^38^:

8 $${\rm{TE}} = - \mathop {\sum }\limits_{a,b} \mathop {\sum }\limits_{P\left( {a,b} \right) \ne O} p\left( {a,b} \right){\mathrm{ln}}\left( {p\left( {a,b} \right)} \right)$$

where each element *p*(*a*, *b*) in an image is the probability of a change from pixel intensity/value *a* to *b*. The natural logarithm ln describes a strict monotonic increase of changes in color with the quantity of measurements. For detailed information confer to Beyenal et al.^39^.

Stated parameters were selected due to their importance regarding the performance of biofilms, e.g. the porosity parameter (Φ_intrinsic_) gives insight into how well substrates could be transported by pores and water channels into the biofilm (which in turn affects $\bar L_F$). Another example: in MFCs (microbial fuel cells), a more flat biofilm that fully covers the anode (SC) of the “battery” is potentially more desired to produce electric currents than a fungli-like structure with a high heterogeneity (TE).

We have to state that researchers have to choose their parameters based on the narrative of the investigation. Typically, all parameters are governed by hydrodynamics, substrate addition and/or uptake, specimens, and the biofilm’s proteins, matrix and structure itself.

An overview of selected parameters is given in Yang et al.^39^.

### Statistical evaluation of biofilm parameters

Grubbs’ tests were performed in Origin (OriginPro, Version 2018G, OriginLab Corporation, Northampton, MA, USA) to identify outliers in each parameter set calculated for each day. Occurring outliers were removed.

Number of replicates *n*, total number of replicates *N*, means and medians as well as standard deviations are provided with the results.

### Reporting summary

Further information on research design is available in the Nature Research Reporting Summary linked to this article.

## Supplementary information

Supplementary Information

Reporting Summary

## Data Availability

The authors declare that all data supporting the findings of this study are available within the paper and its supplementary information files.
