# Supplementary material for: An open-source robotic platform that enables automated monitoring of replicate biofilm cultivations using optical coherence tomography
Source: NPJ Biofilms Microbiomes. 2020 Apr 1;6:18. doi: 10.1038/s41522-020-0129-y (PMC7113294; doi:10.1038/s41522-020-0129-y)
Supplement: Supplementary file 1 — Supplementary Information [file 41522_2020_129_MOESM1_ESM.pdf]

Supplementary Information

More information concerning the EvoBot platform and its structural plans can be found here:

- https://blogit.itu.dk/evoblissproject/
- https://bitbucket.org/afaina/evobliss-software/wiki/Home)

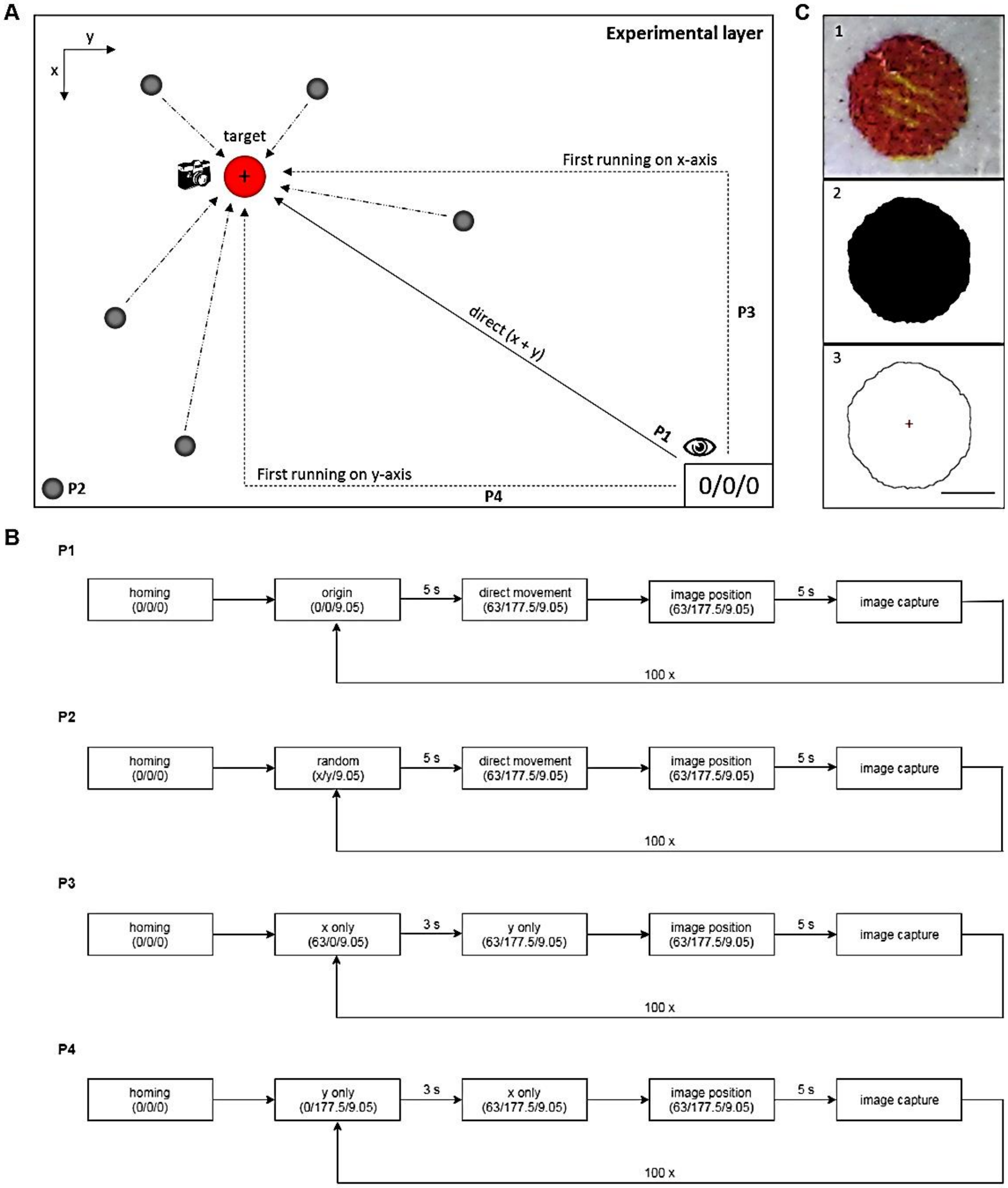

Supplementary Figure 1. **A = Outline of the positioning accuracy test. B = Scheme explaining the steps of the different conditions in positioning experiments P1-P4. C = Image processing steps of captured images: 1 = original; 2 = binary; 3 = analyzed particle; the cross marks the determined Center of Mass. The scale bar has a length of 0.5 mm.**

Supplementary Table 1. Detailed values of the positioning accuracy of the robotic platform in µm. IQR = Interquartile range; $N_{outliers}$ = quantity of outliers; - = no outliers (values outside 1.5 IQR) occurred.

Axis	Experiment	IQR	1.5 IQR	$N_{outliers}$
**x**	**P1**	-1.3 - 1.1	-4.3 - 3.6	-
	**P2**	-5.4 - 8.6	-25.6 - 16.8	6
	**P3**	-1.1 - 1.8	-5.3 - 4.5	2
	**P4**	-1.4 - 2.1	-5.6 - 6.2	3
**y**	**P1**	-4.6 - 4.6	-8.0 - 10.7	-
	**P2**	-6.3 - 5.2	-13.6 - 15.3	-
	**P3**	-6.3 - 7.6	-20.6 - 10.7	2
	**P4**	-2.1 - 1.4	-5.6 - 4.0	2

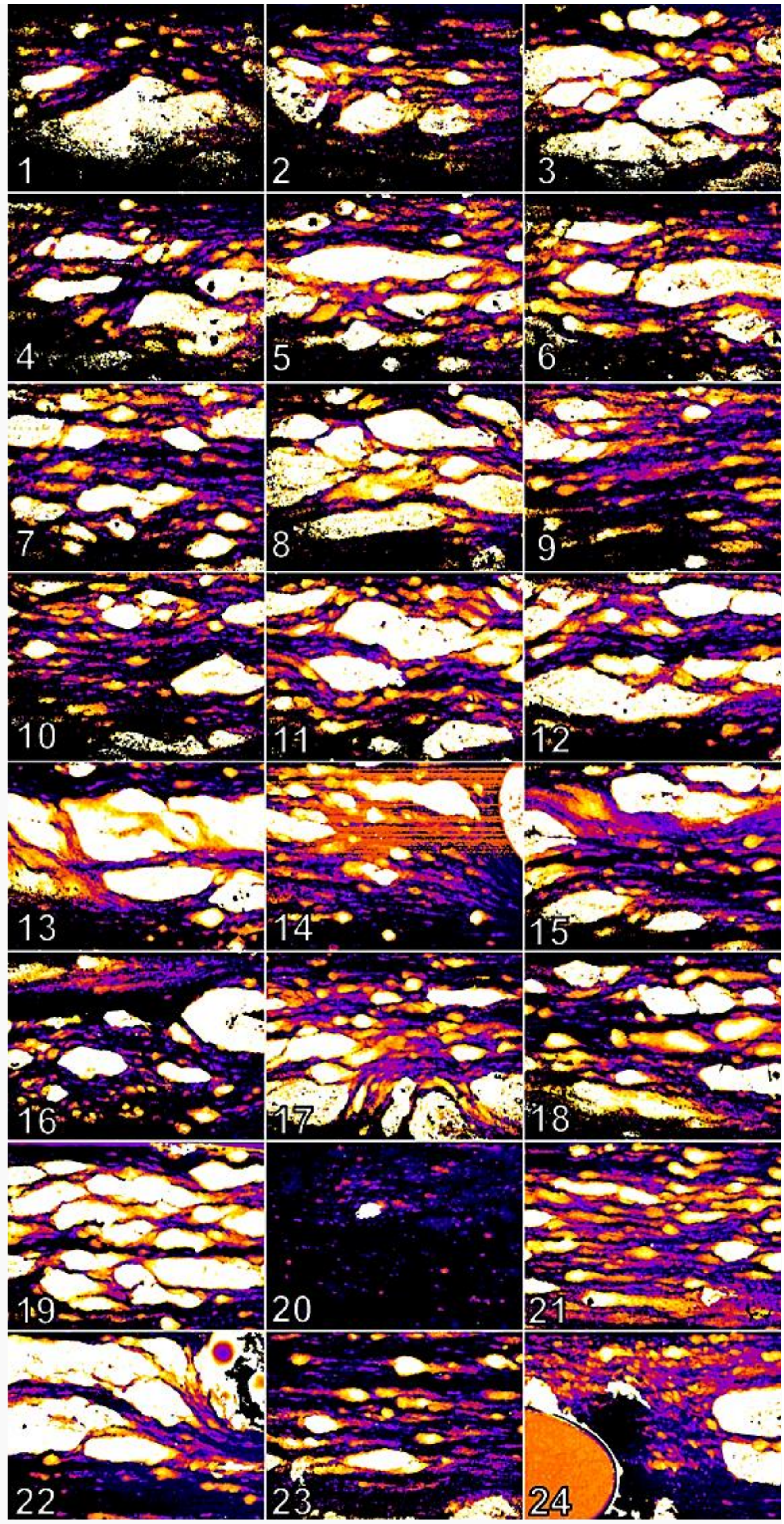

Supplementary Figure 2. Maximum Intensity Projections (MIPs) of all (N = 24) flow cells illustrating day 3 of the experiment. Flow cells 14 and 24 are excluded in calculations due to air bubbles and false reflection signals.

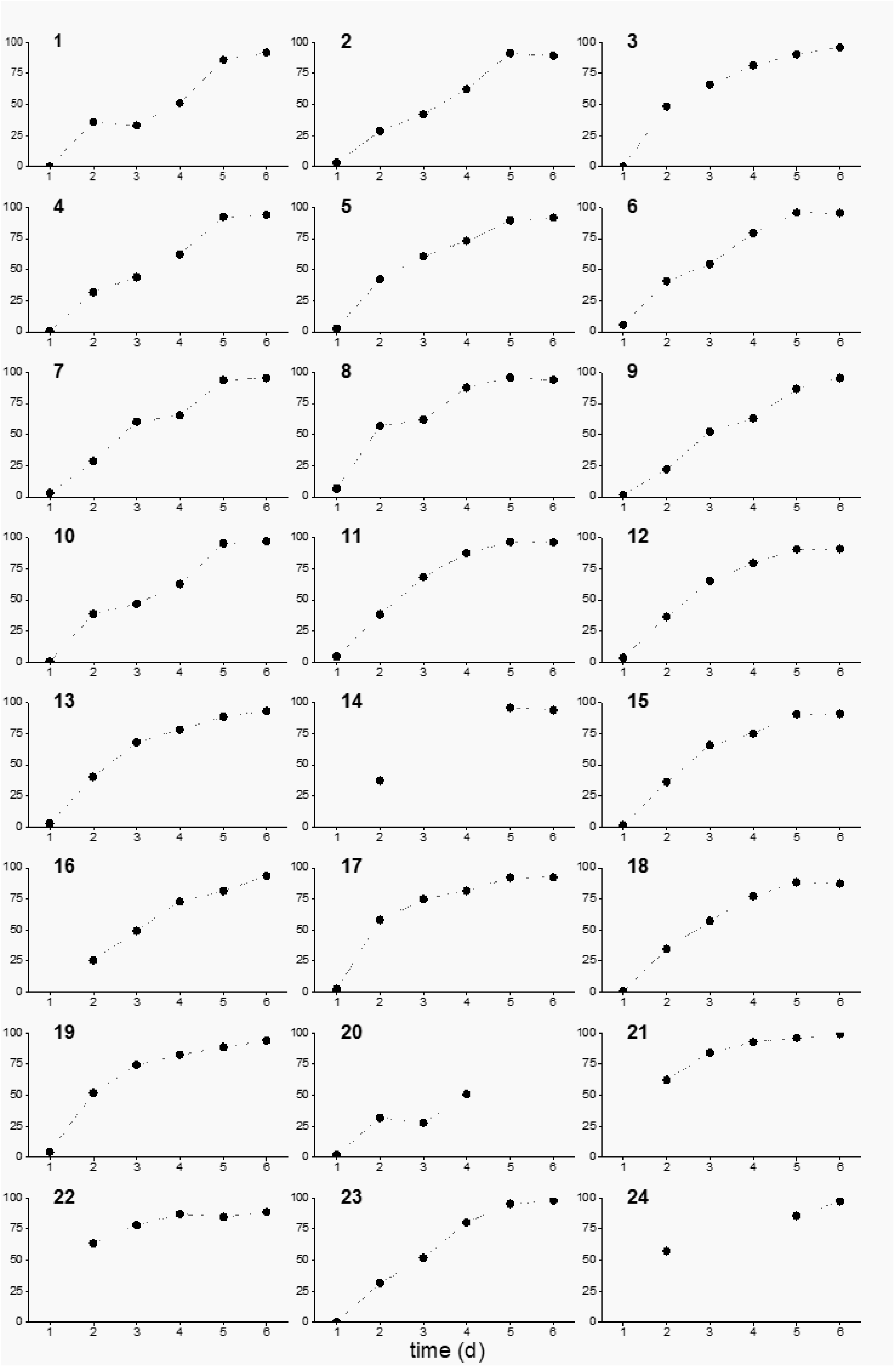

Supplementary Figure 3. Surface coverage SC of all (N = 24) flow cells over the course of the experiment. Dotted lines for better visualization (no data points). Missing values due to air bubbles or outliers are excluded in the diagrams.

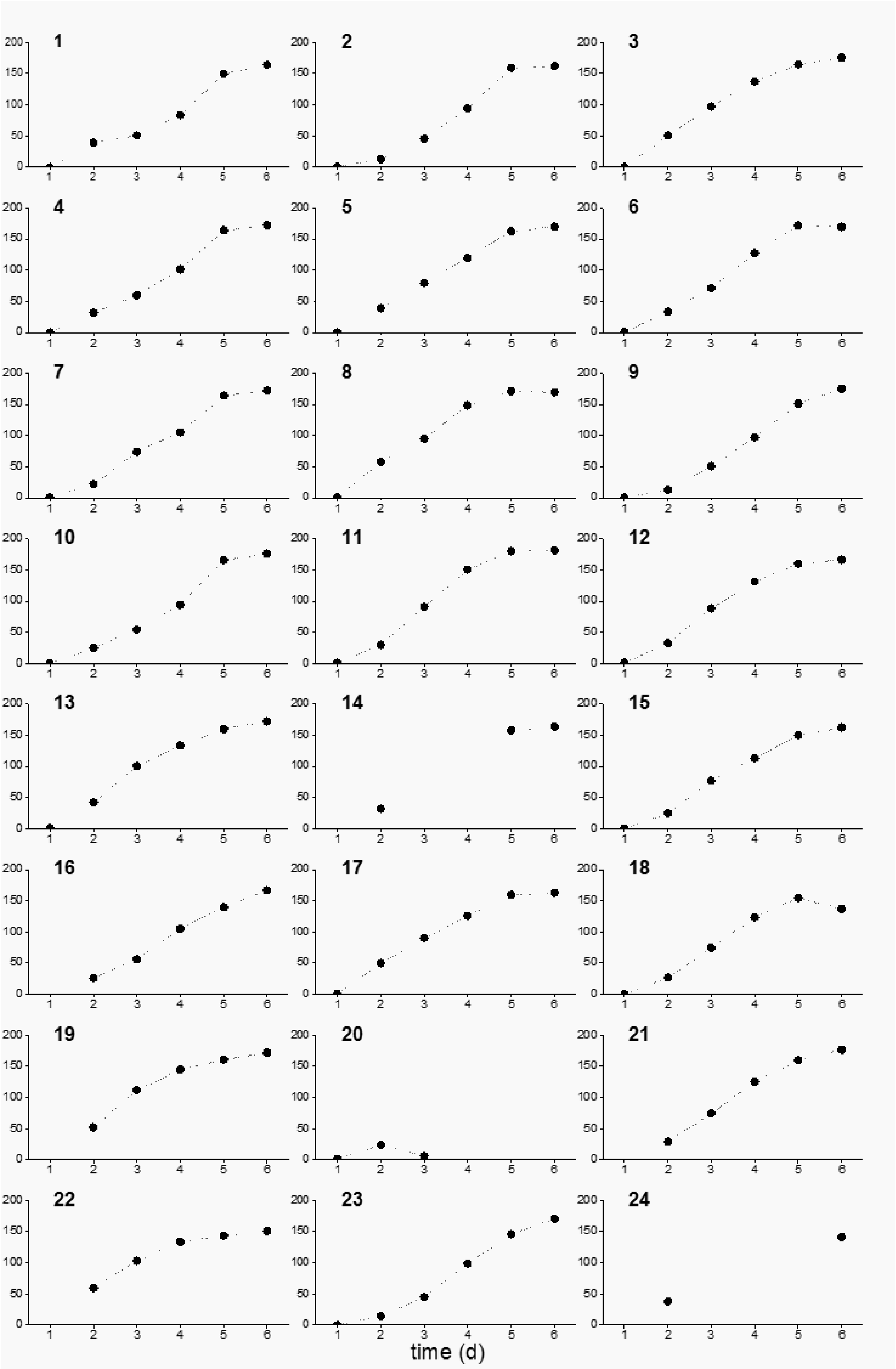

Supplementary Figure 4. Mean biofilm thickness $\bar{L}_F$ of all (N = 24) flow cells over the course of the experiment. Dotted lines for better visualization (no data points). Missing values due to air bubbles or outliers are excluded in the diagrams.

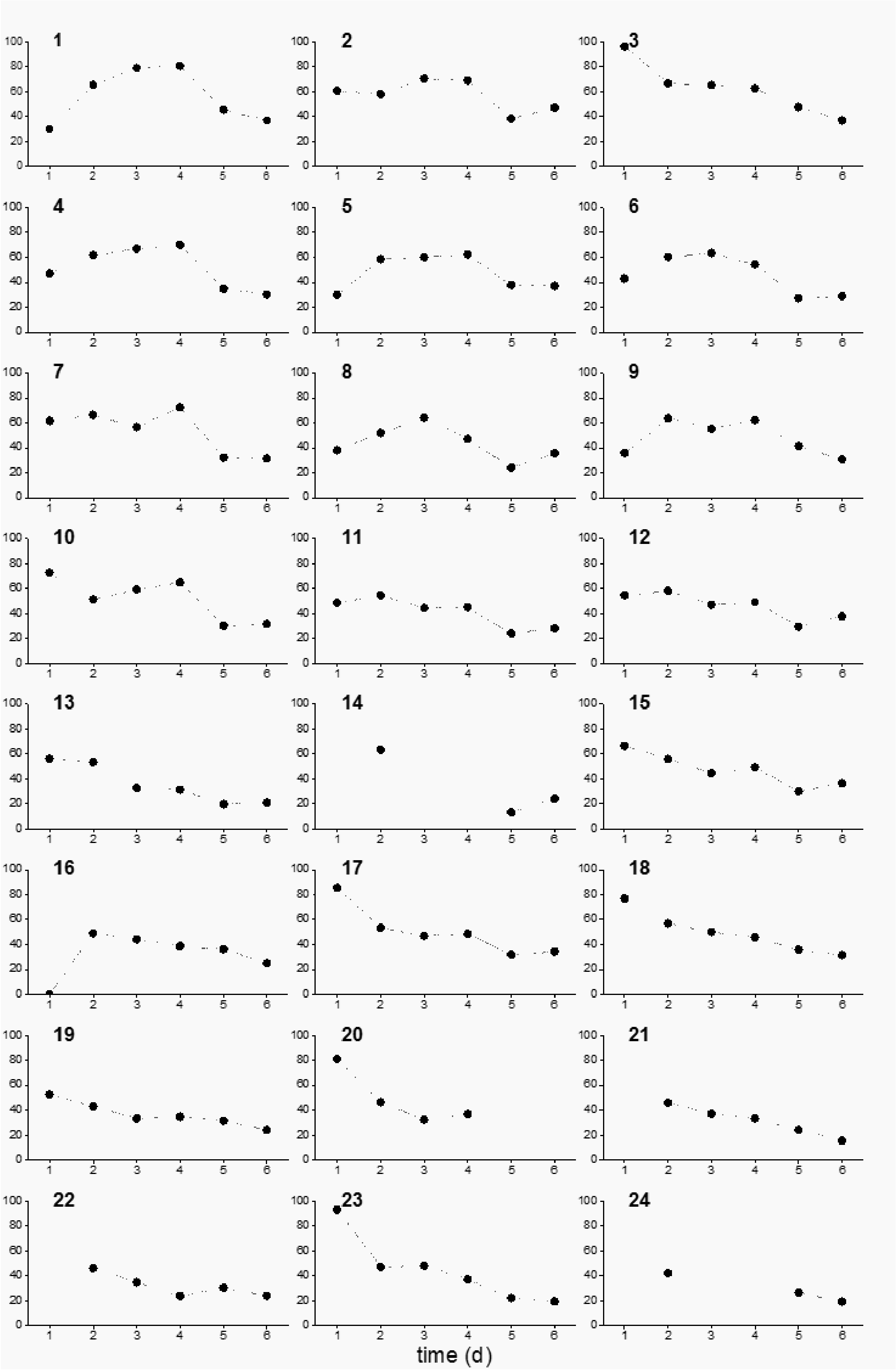

Supplementary Figure 5. Intrinsic biofilm porosity $\Phi intrinsic$ of all (N = 24) flow cells over the course of the experiment. Dotted lines for better visualization (no data points). Missing values due to air bubbles or outliers are excluded in the diagrams.

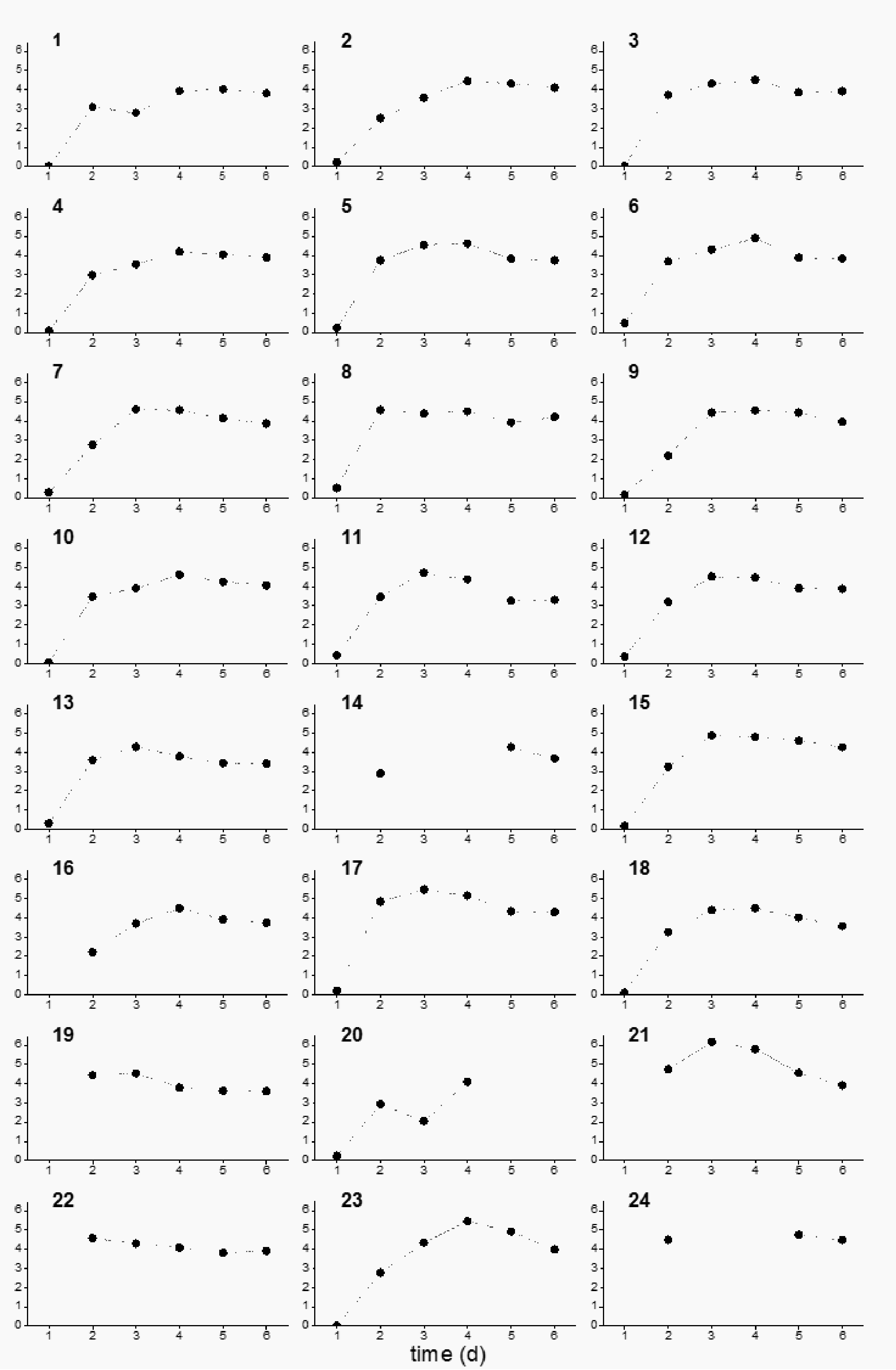

Supplementary Figure 6. Textural entropy TE of all (N = 24) flow cells over the course of the experiment. Dotted lines for better visualization (no data points). Missing values due to air bubbles or outliers are excluded in the diagrams.

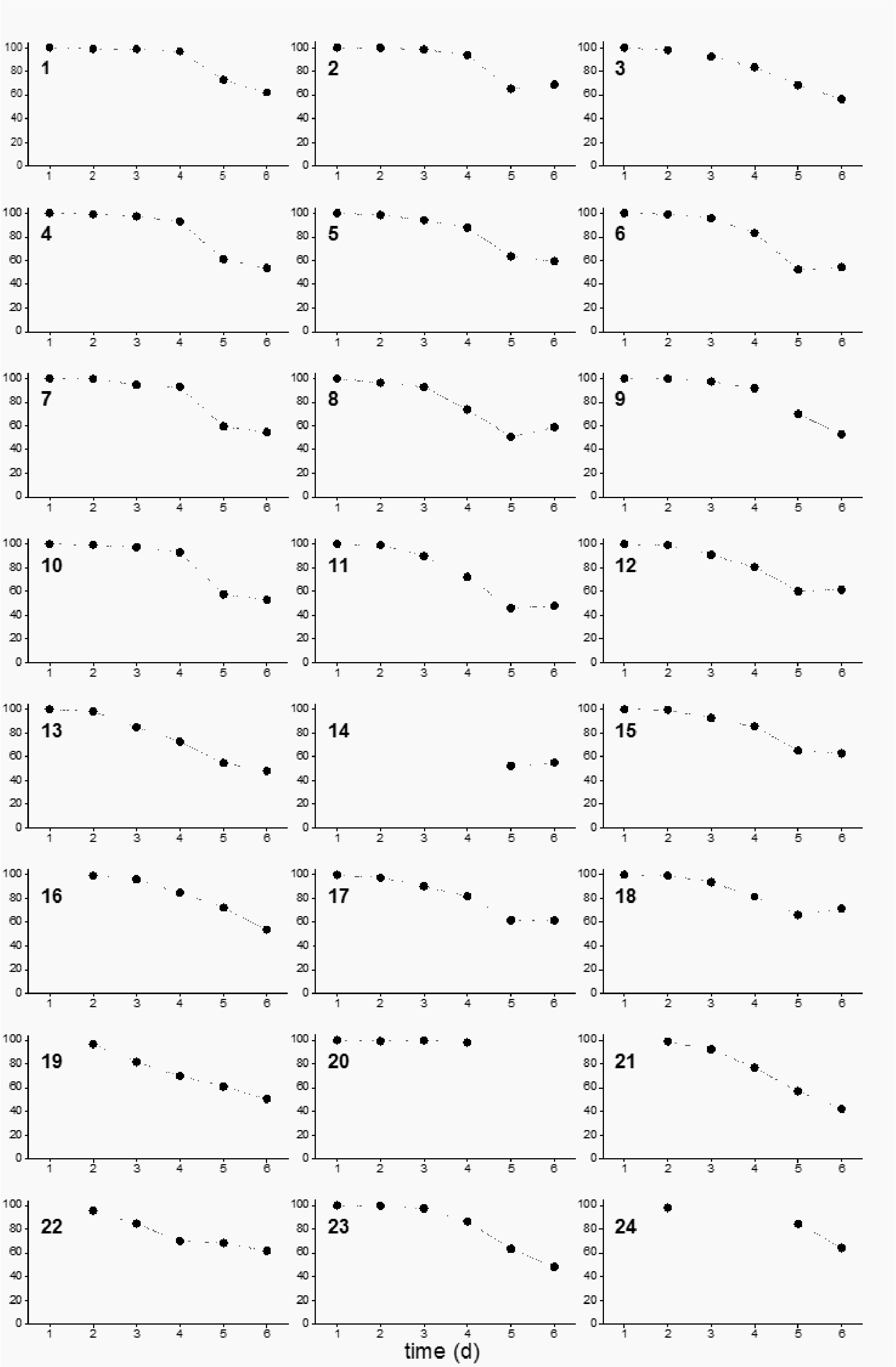

Supplementary Figure 7. Global biofilm porosity $\Phi global$ of all (N = 24) flow cells over the course of the experiment. Dotted lines for better visualization (no data points). Missing values due to air bubbles or outliers are excluded in the diagrams.
